# AUSTRIAN SYNDROME: A RARE CASE REPORT

**DOI:** 10.21010/Ajidv18i1.4

**Published:** 2023-10-20

**Authors:** HASSAN Kamena Mwana-Yile, EJJEBLI Samia, BADI Hanane, BUCUMI Jean Claude, MARHOUM Kamal El Filali

**Affiliations:** 1 Department of Infectious Diseases, Ibn Rochd University Hospital, Faculty of Medicine and Pharmacy, Hassan II University, Casablanca, Morocco; 2 Department of Cardiology, Ibn Rochd University Hospital, Faculty of Medicine and Pharmacy, Hassan II University, Casablanca, Morocco

**Keywords:** Austrian syndrome, *Streptococcus pneumoniae*, Meningitis, Endocarditis

## Abstract

Austrian syndrome is a rare and fatal triad of pneumonia, meningitis and endocarditis caused by Streptococcus pneumoniae, with a mortality rate of 60%. We report a case of Austrian syndrome in a 59-year-old patient, with a history of arterial hypertension on angiotensin 2 receptor antagonist therapy for five years, chronic smoking at 20 packs per year and occasional enolism for fifteen years, presenting with prolonged fever associated with loss of consciousness with no respiratory or cardiac signs, in whom purulent bacterial meningitis with positive Gram stain, infective endocarditis with mitral and aortic localization and interstitial pneumopathy have been demonstrated with negative blood cultures. Although the mortality rate is very high, early management of Austrian syndrome can improve the patient’s quality of life.

## Introduction

*Streptococcus pneumoniae* are gram-positive bacteria that generally colonize the mucosal surfaces of the human upper respiratory tract, with manifestations such as lobar pneumonia, meningitis and endocarditis (Bogaert, *et al*, 2004; Weiser, *et al*, 2018) Austrian syndrome is the triad of pneumococcal pneumonia, meningitis and endocarditis. It was clinically described in 1881 by Osler and subsequently named “Austrian syndrome” in honor of Robert Austrian, who described the affinity of S. pneumoniae for the aortic valve and the simultaneous presence of meningitis in 1957 (Austrian, *et al*,1957). Pneumococcus is responsible for less than 3% of native valve endocarditis, but causes rapid valve destruction (Rakočević et al. 2019). Less than 1% of patients with pneumococcal endocarditis present with the Austrian triad. However, this rare syndrome has a high mortality (Rakočević*, et al*, 2019; Velazquez,*et al*, 2008) but can be well managed when diagnosed early. Few cases of Austrian syndrome have been published, and the majority of published cases have resulted in death. We report a case of Austrian syndrome observed in the Department of Infectious Diseases of the Ibn Rochd University Hospital in Casablanca, Morocco.

### Observation

We report the case of Mr DM, aged 59, who had been treated for hypertension for five years with an angiotensin-2 receptor blocker, was a chronic smoker (20 packs per year) and had been an occasional alcoholic for 15 years. He was admitted with a prolonged fever of 39°C that had been evolving for a month, in a context of weight loss that had not been quantified. The evolution was marked five days before the consultation by the onset of loss of consciousness and persistent fever. On clinical examination, patient is drowsy but rousable (Glasgow14/15) associated with conjunctival and mucous skin pallors, high blood pressure at145/90 mm Hg, tachycardia at 110 beats per minute, tachypnea at 30 cycles per minute and pyrexia at 39°C. Neurological examination revealed no stiff neck or Kerning’s or Burdzinsky’s signs. Cardiovascular examination was normal, with no aortic or mitral murmurs. Pulmonary auscultation revealed crepitating rales at both lung bases. Dermatological examination reveals petechiae on the face, upper limbs and especially the abdomen. Examination of the ear, nose and throat revealed dental caries.

A cerebral CT scan carried out in the presence of fever associated with loss of consciousness showed no abnormalities. A lumbar puncture was performed and cytobacteriological examination of the cerebrospinal fluid (CSF) revealed pleocytosis at 90 with neutrophilic predominance, hyperproteinorachy at 0, 54 mg/dl and normoglycorrhaphy at 0.84 mg/dl, Gram staining showed the presence of gram-positive cocci, while culture was negative after 36 hours, and multiplex Polymerase Chain Reaction (PCR) in cerebrospinal fluid was negative.

**Figure 1:**
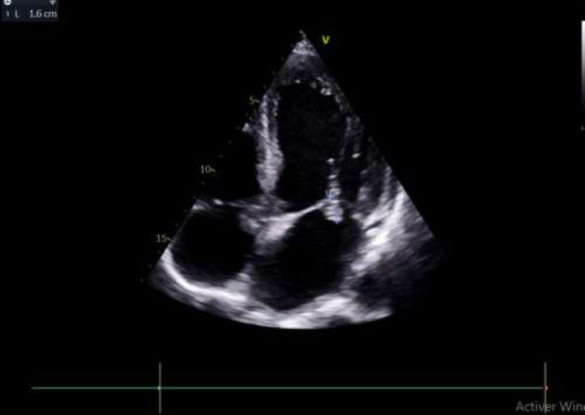
Atrial mitral valve vegetation measuring 19 x 10 mm, with severe mitral insufficiency due to chordal rupture associated with uncompressed left ventricular dilatation to 24 mm and global hypokinesia without cardiac effusion.

**Figure 2:**
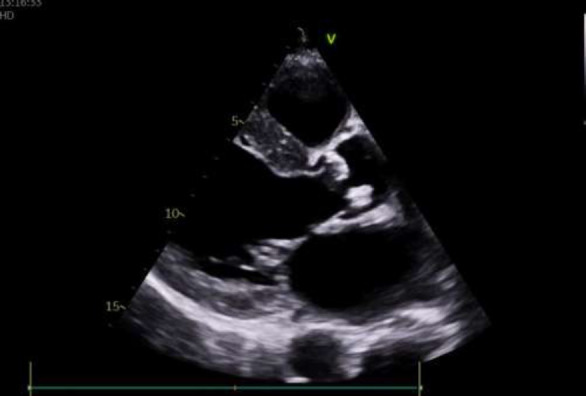
Several vegetations on the tricuspid aortic valve, the largest measuring 9 x 8 mm with severe aortic insufficiency associated with uncompressed left ventricular dilatation to 24 mm and global hypokinesia without cardiac effusion.

In the presence of purulent bacterial meningitis with positive Gram stain and also associated factors of invasive pneumococcal infection with reduced sensitivity to penicillin, a cardiac echocardiography is performed and revealed vegetations on the mitro-aortic valves measuring 19 x 10 mm (Figure 1) and measuring 9 x 8 mm (Figure 2).

**Figure 3:**
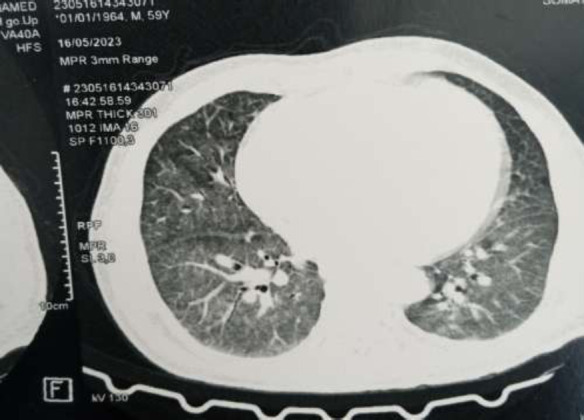
Diffuse bilateral ground-glass plaques on a background of centro-lobular emphysema in favor of diffuse interstitial lung disease with incipient fibrosis

**Figure 4:**
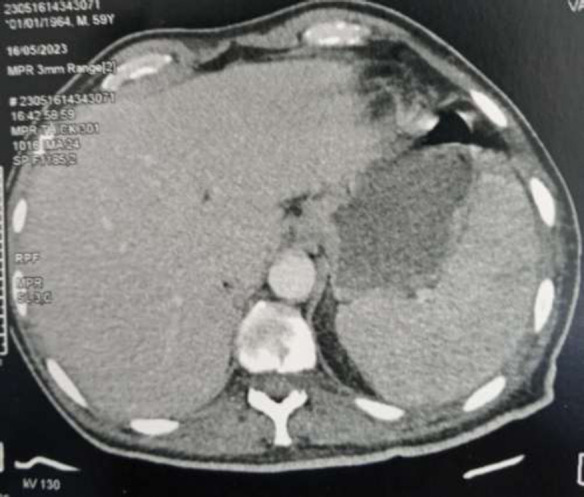
Splenomegaly with area of upper polar hypodensity compatible with the focus splenic infarction, prostatic hypertrophy and a small focus of osteolysis on the vertebral body of the first lumbar vertebra associated with endocarditis.

The electrocardiogram showed tachycardia at 100 bpm and negative lateral T waves. Pulmonary exploration showed diffuse interstitial lung disease with the onset of pulmonary fibrosis and significant cardiomegaly predominating in the left cavities with signs of pulmonary hypertension on chest CT (Figure 3). Abdominal and pelvic splenomegaly with superior polar hypodensity in relation to a splenic infarct focus, prostatic hypertrophy and a small focus of osteolysis on the vertebral body of the first lumbar vertebra (Figure 4).

The emergency laboratory work-up showed an inflammatory syndrome with normocytic normochromic anemia at 6.5 g/dL, hyperleukocytosis at 27760/µl, predominantly neutrophils (25070), thrombocytosis at 595 000 /mm3 and C-reactive protein at 127.9/mL. Renal function was impaired, with creatinine at 16.4 mg/dL, glomerular filtration rate at 45 ml/minute and urea at 1.14 mg/dl. HIV-1 and HIV-2 serology were negative.

Three blood cultures taken before the first dose of ceftriaxone were sterile. Cytobacteriological examination of urine after three days of antibiotic therapy revealed leukocyturia at 11,000/mL and hematuria at 36,000/mL.

After lumbar puncture and before laboratory examinations, treatment with third-generation cephalosporin was initiated at a meningeal dose of 100 mg/kg/d, i.e. 6,000 mg in two infusions.

Once purulent gram-positive bacterial meningitis had been confirmed and mitro-aortic valve vegetations identified, vancomycin and gentamicin were introduced at doses adjusted to renal function : 500 mg in two infusions over 24 hours and 160 mg intravenously over 36 hours.

Symptomatic treatment was combined with antibiotics, including diuretics to treat lesional pulmonary edema, prophylactic low-molecular-weight heparin and high-concentration mask oxygen therapy.

The evolution was marked three days after antibiotic treatment by thermal lysis and regression of hyperleukocytosis to 13470/µl and CRP to 88 mg/L. at five days of treatment.

The patient died in respiratory distress on a high-concentration oxygen mask on the fifteenth day of treatment, before being transferred to cardiovascular surgery for valve replacement.

## Discussion

The risk factors for invasive penicillin-resistant pneumococcal infection are many, including alcoholism and otorhinolaryngology infection. Although blood cultures are negative, the notion of alcohol consumption and otolaryngological infection, as well as the identification of gram-positive cocci on direct examination of cerebrospinal fluid, may point to *Streptococcus pneumoniae*.

In 5 to 10% of cases of endocarditis, blood cultures remain negative (Baddour, *et al*, 2015). However, once the bloodstream has been invaded, the release of inflammatory mediators triggered by the infection facilitates the passage of pneumococcus across the blood-brain barrier and the development of meningitis (Mook-Kanamori, *et al*, 2011).

In all cases of purulent bacterial meningitis, cardiac ultrasound should be performed as soon as possible to detect infective endocarditis, as the difficulty of recognizing cardiac involvement early (absence of stigmata and symptoms already explained by lung and central nervous system involvement) contributes to the poor prognosis of Austrian syndrome (Giancarlo, *et al;* 2011).

Similarly, although *Streptococcus pneumoniae* remains the most common bacterium causing community meningitis in adults, with high mortality (25%) and morbidity rates despite adequate antibiotic or corticosteroid treatment (Atkinson, *et al*, 2009; Auburtin, *et al*,2002), *Streptococcus pneumoniae* endocarditis is rare.

In a retrospective study of 80 cases of pneumococcal meningitis in intensive care units, only six patients developed endocarditis (Atkinson, *et al*, 2009).

The lungs would be the most frequent microbial portal of entry leading to the development of Austrian syndrome, followed by endocarditis and meningitis (Siles Rubio, *et al*, 1998; Munoz, *et al*, 1999), respectively. Whatever the route of entry, the most common predisposing factors are alcohol consumption, male gender and advanced age (Gransden, *et al*, 1985; Baig, *et al*, 2012).

The patient in our clinical case had alcoholism and male gender as predisposing factors. It has previously been reported that concomitant infective endocarditis is present in 30% of cases of spondylodiscitis (Behmanesh, *et al*, 2019; Carbone, *et al*, 2020). *Streptococcus pneumoniae* is still a major cause of pneumonia, with bacteremia occurring in around 20% of case (Bordon, *et al*, 2015).

## Conclusion

Austrian syndrome is a rare but serious pathological association with a mortality rate of 60% if not diagnosed at an early stage. Therefore, in patients with predisposing risk factors, even in the absence of a heart murmur, clinicians should suspect it as early as possible for better management.

### Conflict of Interest Declaration:

The authors declare that there is no conflict of interest associated with this study.

### Consent of the family member

We obtained the consent of the patient’s wife for the writing of this clinical case and the submission of the clinical case study. We masked the patient’s name on the scan and ultrasound images.

**List of Abbreviations:**
PCR: Polymerase Chain Reaction
CRP: C-reactive protein
CSF: Cerebralspinal fluid
CT-scan: computed tomography
